# Comparative study on clinicopathological features and prognosis of IgA vasculitis nephritis and IgA nephropathy in children

**DOI:** 10.1186/s12887-023-04243-3

**Published:** 2023-08-24

**Authors:** Yan Lv, Rui Fu, Xiao-Jie Peng, Ying Wang, Ting-Ting Yin, Yan-Qing Deng

**Affiliations:** 1 Department of Nephrology, Jiangxi Provincial Children’s Hospital, Nanchang, China; 2 Nanchang University, Nanchang, Jiangxi Province China

**Keywords:** IgA vasculitis nephritis, IgA nephropathy, SQC, Kidney prognosis, Children

## Abstract

**Background:**

IgA vasculitis nephritis (IgAVN) and IgA nephropathy (IgAN) share several clinical and pathological characteristics, though distinctions also exist. Their interrelation, however, remains undefined. This study investigates the clinicopathological divergences and prognostic disparities in pediatric patients with IgAVN and IgAN.

**Methods:**

Our study encompasses 809 pediatric patients with IgAVN and 236 with IgAN, all of whom underwent kidney biopsy. We utilized the Semiquantitative Classification (SQC) scoring system to juxtapose the pathologies of the two conditions, and performed a COX regression analysis to examine factors influencing their prognoses.

**Results:**

Both patient groups demonstrated a predominance of males. A seasonality was observed, with a higher incidence of IgAN in the summer, and IgAVN in the fall (*P* < 0.0001). Patients with IgAN exhibited more severe tubulointerstitial injury, higher chronicity index, and total biopsy scores compared to those with IgAVN (*P* < 0.0001). Mesangial deposition intensity of complement C3, and the rate of pure IgA deposition, were found to be greater in patients with IgAVN compared to those with IgAN (*P* < 0.0001). The intensity of IgA deposition was also significantly higher in IgAVN patients (*P* = 0.003). IgAVN demonstrated a superior prognosis, with a higher rate of kidney remission (*P* < 0.0001). COX regression analysis indicated that interstitial fibrosis, as identified in the SQC pathology system, was associated with the prognosis of both conditions. Furthermore, the findings suggest that IgA deposition levels (IgA +  + and IgA +  + +) could potentially influence the prognosis of IgAVN.

**Conclusions:**

Compared to IgAVN, IgAN manifests more severely with regard to renal impairment, interstitial damage, and prognosis. The disparities in immune complex deposition levels and locations within the kidneys support the hypothesis of IgAVN and IgAN as distinct diseases. Interstitial fibrosis may serve as a key pathological indicator within the SQC system associated with kidney prognosis in children with IgAVN and IgAN. The degree of IgA deposition could also be linked with the prognosis of IgAVN.

## Introduction

IgA vasculitis (IgAV), a prominent type of vasculitis among pediatric populations, is marked by its capacity to impact small blood vessels in multiple organs, including the skin, and is distinguished by the presence of immunofluorescent IgA deposits. This condition is specifically termed IgA vasculitis nephritis (IgAVN) when it affects the kidneys [1]. On the other hand, IgA nephropathy (IgAN) is one of the most common primary glomerular diseases in children, often characterized by recurrent proteinuria and hematuria [2]. IgAVN and IgAN, both IgA-mediated autoimmune disorders with similar predisposing factors, display characteristic glomerular IgA deposits, predominantly composed of IgA1 [2].

The Oxford classification for IgAN, first introduced in 2009, identified four histopathologic features—mesangial hypercellularity (M), endocapillary hypercellularity (E), segmental glomerulosclerosis (S), and tubular atrophy and interstitial fibrosis (T)—as determinants of kidney prognosis independent of clinical features [3, 4]. By 2016, the addition of crescents (C) to the classification was recommended by the IgAN working group [5]. The Oxford classification has been widely utilized and its prognostic value affirmed in numerous studies [6–8]. However, it inadequately addresses the type and severity of tubulointerstitial inflammation, which is a potentially overlooked prognostic factor. Asrar et al. [9] found that, upon excluding patients with severe pathological changes (T2), tubulointerstitial inflammatory changes were present in 90.2% of the remaining 247 patients, with moderate inflammatory changes being the most prevalent (49.4%). Importantly, the type and severity of tubulointerstitial inflammation correlated strongly with M, E, T, and C. Considering the predictive value of the MEST-C system for kidney regression in IgAN, tubulointerstitial inflammation may hold significant application potential. Similarly, Rankin et al. [10] discovered an independent association between tubulointerstitial inflammation and the prognosis of IgAN, surpassing even the predictive value of the MEST-C system.

In 2017, Koskela et al. [11] proposed a modified semiquantitative scoring system (SQC) for IgAVN, which evaluated kidney tissue via four aspects—glomerular, tubular, interstitial, and capillary changes. It distinguished between active and chronic lesions and scored each pathological index based on lesion severity. The authors reported that the SQC's predictive power for kidney prognosis was comparable to the International Study of Kidney Disease in Children (ISKDC) system when applied to IgAVN. In light of the existing clinical, immunological, and histological parallels between the two disorders and shared etiological factors [12, 13], as well as the effective application of the Oxford classification in recent IgAVN studies [14–18], we sought to assess kidney pathology in IgAVN and IgAN using the SQC scoring system. Our focus was to explore the impact of the SQC pathology index on prognosis and identify the clinicopathological and prognostic similarities and differences. This would provide a stronger foundation for their clinical differentiation.

## Methods

### Patients and inclusion criteria

**Fig. 1:**
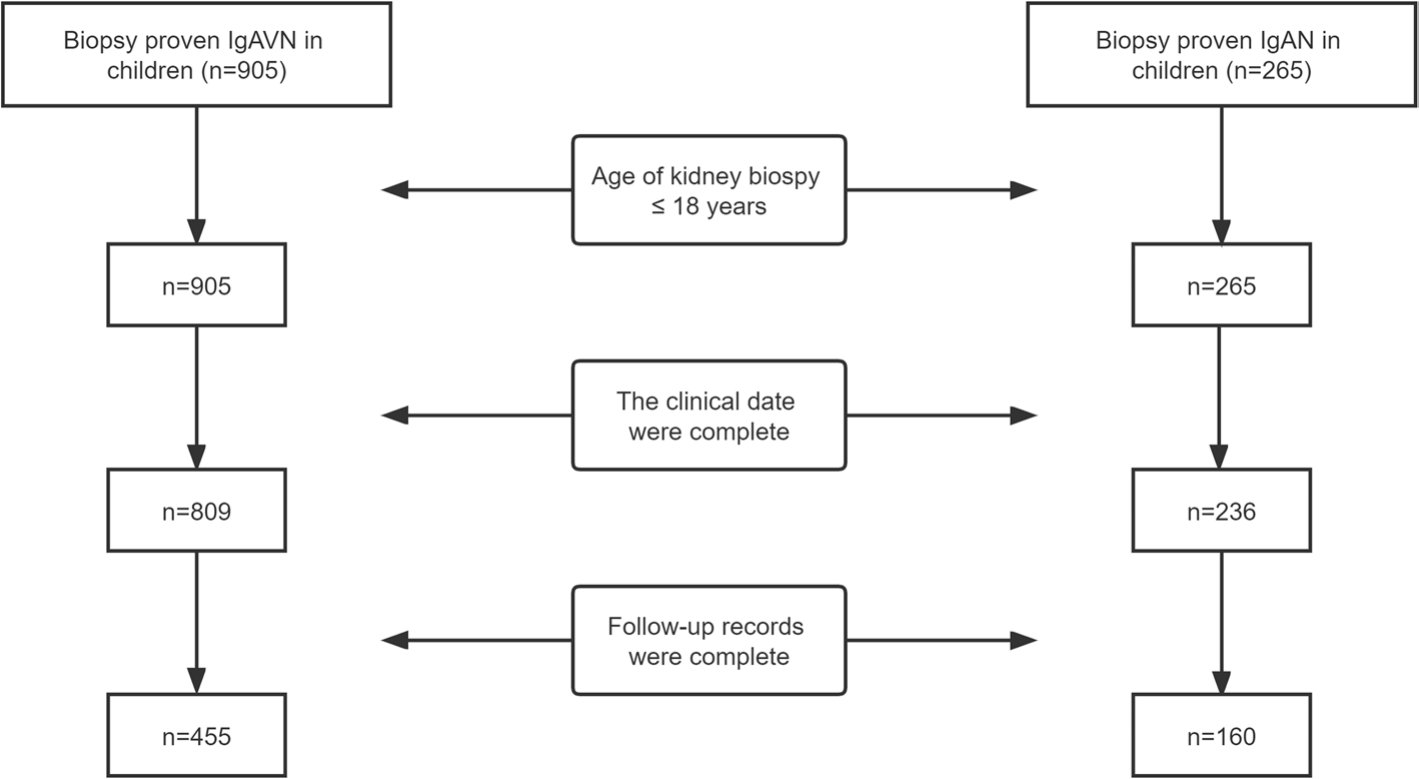
Flow chart of case screening in IgA vasculitis nephritis (IgAVN) and IgA nephropathy (IgAN) groups

Children with IgAVN and IgAN who underwent kidney biopsy in Jiangxi Provincial Children’s Hospital were enrolled from January 1, 2006, to December 31, 2021, in this retrospective cohort study. We gathered data on all kids in hospitals when they had their first kidney biopsies, such as clinical and kidney pathology biopsy data, and thoroughly analyzed the information. At the same time, we collected the last follow-up records of 455 children with IgAVN and 160 children with IgAN and analyzed their prognosis (Fig. 1). Diagnostic criteria for IgAVN: meets EULAR/PRINTO/PRES diagnostic criteria [19]. IgAN diagnostic criteria: kidney biopsy confirmed that predominant mesangial deposition of IgA, those with secondary causes of mesangial IgA deposits were excluded.

### Clinical data

All clinical data were gathered during the child's first kidney biopsy. The medical records of the enrolled children's visits were reviewed to acquire clinical data during kidney biopsy and follow-up. Gender, age, time from onset to kidney biopsy, time from first kidney biopsy to the most recent clinic visit, the season of onset, clinical presentation, blood pressure, urinary routine, and kidney histopathology results were among the information collected. Hematoxylin–eosin, PAS, PAM, Masson staining, and immunofluorescent labeling of kidney tissue sections, including IgA, IgM, IgG, C3, C4, C1q, and FRA, were among the staining procedures used for kidney tissue specimens. Hematuria was divided into microscopic hematuria and gross hematuria. Hypertension was defined as average systolic or diastolic blood pressure greater than or equal to the 95th percentile for age, gender, and height.

### Pathology

Criteria for a kidney biopsy was IgAVN or IgAN patients who had active urinary sediments (proteinuria or hematuria). Proteinuria was defined as a positive dipstick reading of ≥ 1 + , urinary protein ≥ 0.2 g/m2/day. Hematuria was defined as five or more red blood cells per high-power field in a properly collected and centrifuged urine specimen. Pathological grading: Referring to the grading of Bohle et al. [20], the kidney interstitial fibrosis and tubular atrophy were classified into (-) to (+ +  + +) grades according to their degree of atrophy. Semiquantitative classification (SQC): a modified semiquantitative score developed by Koskela et al. [11] evaluates kidney tissue in four ways: glomerular, tubular, interstitial, and capillary changes, identifying active and chronic index, and assigns a pathological score to each pathological index based on the severity of the lesion, with active lesions receiving nine points, chronic lesions were receiving sixteen points, and focal or diffuse mesangial proliferation receiving one point (total 26 points). Two pathologists, who have experience evaluating kidney pathology specimens, reevaluated each pathology sample without any clinical information.

Immunopathological typing: Ten types of immune complexes were classified according to the type of immune complexes deposited in the glomerulus: ①Simple IgA deposition; ②IgA + C3; ③IgA + C4; ④IgA + IgM; ⑤IgA + IgG; ⑥IgA + IgM + C3; ⑦IgA + IgG + C3; ⑧IgA + IgM + IgG; ⑨IgA + IgM + IgG + C3; ⑩IgA + IgM + IgG + C3 + C4. The degree of deposition of IgA (1–4 +), C3 (0–3 +) was defined as follows: -; negative, 1 + ; weak but definite staining, 2 + ; moderate, 3 + ; strong staining and 4 + ; bright staining. Concerning C1q, C4 and FRA, the presence or absence of the staining was determined.

### Treatment

Steroids combined with RARS were used in children with combined proteinuria at a starting steroid dose of 1.5–2 mg/kg/d, and steroids combined with immunosuppressants were used in children with proteinuria levels greater than 50 mg/kg/d; the commonly used immunosuppressant is cyclophosphamide. We recommend methylprednisolone pulse in cases of crescents with more than 25% of glomeruli.

### Kidney prognosis

Grade A (complete remission): normal kidney function, without proteinuria or hematuria; Grade B (partial remission): persistent proteinuria (< 1.0 g/d), and/or hematuria (≥ 3 red blood cells/high power field) without kidney insufficiency; Grade C (no remission): persistent proteinuria (≥ 1.0 g/d), and/or hematuria with moderate kidney failure (< 30% decrease in the eGFR from the baseline); Grade D (kidney failure): ≥ 30% decline in the eGFR from the baseline, End-stage kidney disease (ESRD) or death. IgAVN and IgAN pediatric patients' kidney remission rates were compared using a plot of the Kaplan–Meier survival analysis of survival curves.

### Statistical analysis

Data analysis was executed using SPSS for windows version 26 (IBM Corporation, Armonk, NY). Normally distributed data were expressed as the mean ± standard deviation (SD), and nonparametric data were expressed as the median and interquartile (IQR) range. The count data were expressed as percentages, and Chi-squared Test or Fisher's exact probability test was used for group comparison. Nonparametric variables were compared with the Kruskal–Wallis test. Kidney survival rates were analyzed by the Kaplan–Meier method and compared by log-rank test. The Cox proportional hazards regression model was conducted on univariable and multivariable analyses of SQC classification in the IgAVN and IgAN children. Univariable Cox regression analysis was performed for each pathological variable and deposition intensity of IgA and C3. Multivariable Cox regression analysis was performed to appraise the influence of SQC system and deposition intensity of IgA and C3 on the kidney outcome. Hazard ratio (HR) with 95% confidence interval (CI) for each variable was estimated. All probabilities were two-tailed, and *P* < 0.05 was considered statistically significant.

## Results

### Comparison of clinical data

Clinical baseline characteristics are provided in Table 1. IgAN and IgAVN showed higher prevalence during the spring and winter seasons, while IgAVN was more common in the autumn (*P* < 0.0001). As for clinical symptoms, IgAN presented a higher incidence of gross hematuria, whereas IgAVN was more likely to manifest as isolated microscopic hematuria (*P* < 0.0001). Conversely, IgAVN was associated with a higher incidence of hypertension (*P* = 0.005).

**Table 1:** Comparison of the clinical data of IgA vasculitis nephritis (IgAVN) and IgA nephropathy (IgAN)

	IgAVN (*n* = 809)	IgAN (*n* = 236)	*P*
Male gender (%)	479 (59.2)	167 (70.8)	0.001
Age (years)			
Overall (years, IQR)	8.5 (7.0,11.0)	9.0 (7.0,11.0)	
Grouping			0.085
1–3	8 (1.0)	5 (2.1)	
4–6	174 (21.5)	52 (22.0)	
7–9	310 (38.3)	69 (29.2)	
10–12	223 (27.6)	82 (34.8)	
13–14	72 (8.9)	23 (9.8)	
15–18	22 (2.7)	5 (2.1)	
Time from onset to kidney biopsy (months, IQR)	1.0 (0.57,1.3)	1.0 (0.5,2.0)	0.008
Onset seasons (n, %)			< 0.0001
Spring	198 (24.5)	70 (29.6)	
Summer	92 (11.4)	45 (19.1)	
Autumn	226 (27.9)	33 (14.0)	
Winter	293 (36.2)	88 (37.3)	
Clinical manifestation (n, %)			
Normal urine test	16 (2)	2 (0.8)	
Simple microscopic hematuria	635 (78.5)	99 (41.9)	< 0.0001
Gross hematuria	170 (21)	135 (57.2)	< 0.0001
Hypertension	232 (28.7)	46 (19.5)	0.005

*IQR* Interquartile range

### Comparison of kidney pathological changes

IgAN was found to inflict more severe kidney tubulointerstitial injury compared to IgAVN (*P* < 0.0001). According to the SQC, cellular crescents were more prevalent in IgAVN glomerular lesions (*P* = 0.001). Additionally, statistically significant differences were observed between IgAVN and IgAN concerning the pathological scores for complete tubular atrophy (*P* < 0.0001), interstitial fibrosis (*P* = 0.002), interstitial or periglomerular inflammation (*P* = 0.002), capillary arteriosclerosis or arterial inflammation (*P* < 0.0001), and diffuse mesangial proliferation (*P* < 0.0001). IgAN demonstrated a higher chronicity index and total biopsy score compared to IgAVN (*P* < 0.0001). Further details can be found in Table 2.

**Table 2:** Comparison of pathological changes on kidney biopsy in IgA vasculitis nephritis (IgAVN) and IgA nephropathy (IgAN)

Pathological changes	IgAVN (*n* = 809)	IgAN (*n* = 236)	*P*
Kidney tubular-interstitial grading (n, %)			< 0.0001
-	164 (20.3)	40 (17.0)	
+	267 (33.0)	31 (13.1)	
+ +	314 (38.8)	36 (15.3)	
+ + +	64 (7.9)	128 (54.2)	
+ + + +	0 (0)	1 (0.4)	
Modified SQC			
Glomerular changes			
Lobulation (Active)	0 (0,0)^a^	0 (0,0)^a^	
Mesangial proliferation (Active)	1 (1,1)^a^	1 (1,1)^a^	
Crescents			
Cellular (Active)	0 (0,0)^b^	0 (0,0)^b^	0.001
Fibrous (Chronic)	0 (0,2)^b^	0 (0,2)^b^	
Adhesions (Chronic)	0 (0,1)^b^	0 (0,0)^b^	
Fibrinous thrombosis (Active)	0 (0,0)^b^	0 (0,0)^b^	
Global sclerosis (Chronic)	0 (0,0)^b^	0 (0,1)^b^	
Segmental sclerosis (Chronic)	0 (0,0)^c^	0 (0,0)^c^	
Tubular changes			
Thickening of the basement membrane (Chronic)	0 (0,0)^a^	0 (0,0)^a^	
Complete atrophy (Chronic)	1 (0,1)^a^	1 (1,1)^a^	< 0.0001
Tubular dilatation (Active)	0 (0,0)^a^	0 (0,0)^a^	
Interstitial changes			
Fibrosis (Chronic)	0 (0,1)^a^	1 (0,1)^a^	0.002
Inflammation OR periglomerular inflammation (Chronic)	1 (0,1)^a^	1 (1,1)^a^	0.002
Capillary changes			
Arteriosclerosis OR arterial inflammation (Chronic)	0 (0,0)^a^	0 (0,1)^a^	< 0.0001
Focal or diffuse mesangial proliferation	0 (0,1)^d^	1 (1,1)^d^	< 0.0001
Activity index	1 (1,2)	1 (1,2)	0.064
Chronicity index	3 (1,6)	4 (2,6)	< 0.0001
Total biopsy score	5 (3,8)	6 (5,9)	< 0.0001

*SQC* Semiquantitative classification

^a^0 = absent; 1 = present

^b^0 = 0% of glomeruli affected; 1 = 0–5% of glomeruli affected; 2 = 5–10% of glomeruli affected; 3 =  > 10% of glomeruli affected

^c^0 = 0% of glomeruli affected; 1 = 0–5% of glomeruli affected; 2 =  > 5% of glomeruli affected

^d^0 = 0 for focal, 1 for diffuse

### Comparison of immunopathology data

IgAVN frequently exhibits IgA +  +  + deposits, whereas IgA +  + deposition is more common in IgAN, and the difference is statistically significant (*P* = 0.003). While both IgAVN and IgAN demonstrate C3 + deposition dominance, the proportion of C3 + is considerably higher in IgAN (*P* < 0.0001). Moreover, IgA deposits are typically localized to the mesangium in IgAVN, whereas IgAN displays a higher propensity for IgA deposition within both the mesangium and the segmental loop (*P* < 0.0001). Further details can be found in Table 3.

**Table 3:** Comparison of immunopathological data between IgA vasculitis nephritis (IgAVN) and IgA nephropathy (IgAN)

Immune complexes	IgAVN (*n* = 809)	IgAN (*n* = 236)	*P*
Type of deposition (n, %)			0.804
IgA	68 (8.4)	37 (15.7)	
IgA + C3	84 (10.4)	11 (4.7)	
IgA + C4	4 (0.5)	1 (0.4)	
IgA + IgM	102 (12.6)	26 (11.0)	
IgA + IgG	15 (1.8)	1 (0.4)	
IgA + IgM + C3	240 (29.7)	70 (29.6)	
IgA + IgG + C3	47 (5.8)	11 (4.7)	
IgA + IgM + IgG	40 (4.9)	7 (3.0)	
IgA + IgM + IgG + C3	197 (24.4)	68 (28.8)	
IgA + IgM + IgG + C3 + C4	12 (1.5)	4 (1.7)	
IgA deposition intensity (n, %)			0.003
IgA1 +	111 (13.7)	28 (11.9)	
IgA2 +	316 (38.8)	131 (55.5)	
IgA3 +	380 (47.0)	76 (32.2)	
IgA4 +	2 (0.2)	1 (0.4)	
C3 deposition intensity (n, %)			< 0.0001
C3-	225 (27.8)	71 (30.1)	
C3 +	294 (36.4)	125 (53.0)	
C3 + +	247 (30.5)	40 (16.9)	
C3 + + +	43 (5.3)	0 (0)	
C1q (n, %)	75 (9.3)	28 (11.9)	
C4 (n, %)	34 (4.2)	7 (3.0)	
FRA (n, %)	537 (66.4)	165 (69.9)	
Sites of IgA deposition (n, %)			< 0.0001
Pure-mesangium	585 (72.3)	117 (49.6)	
Pure-segment loop	22 (2.7)	9 (3.8)	
Mesangium + segment loop	202 (25.0)	110 (46.6)	

### Comparison of treatments and kidney prognosis of IgAVN and IgAN

**Fig. 2:**
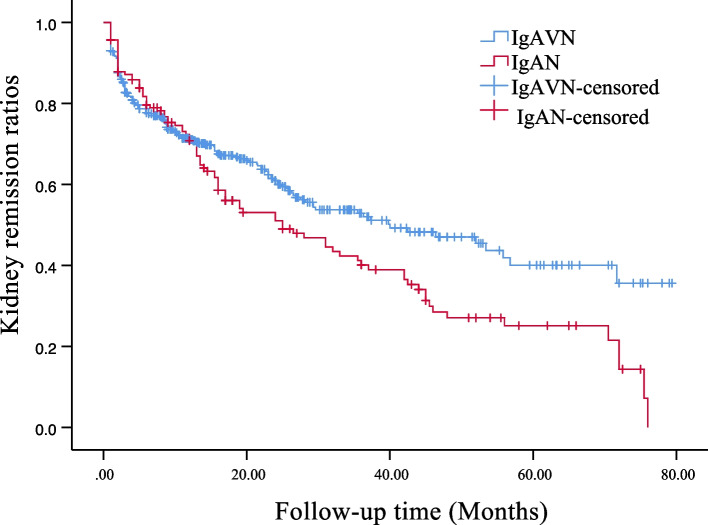
Kaplan–Meier analysis of kidney remission rates in IgA vasculitis nephritis (IgAVN) and IgA nephropathy (IgAN) (Log-rank test, *P* = 0.017)

The therapeutic measures and kidney prognosis are elaborated in Table 4. No significant distinction exists in treatment between IgAVN and IgAN. Both conditions receive more Renin-Angiotensin System Blocker (RASB) therapy, with a relatively low rate of immunosuppressant usage, implying a preponderance of patients with mild disease in our study cohort. The 1-, 3-, and 5-year kidney remission rates were markedly lower in the IgAN group compared to the IgAVN group (70.8% vs. 71.1%, 40.1% vs. 52.0%, and 25.1% vs. 40.0%; Log-rank test, *P* = 0.017) as depicted in Fig. 2. To investigate the impact of the Semiquantitative Scoring (SQC) system's pathological indicators and the intensity of immune complex IgA and C3 deposition on kidney remission rates of IgAVN and IgAN, Cox proportional hazard models were established, as shown in Table 5.

**Table 4:** Comparison of kidney prognosis in IgA vasculitis nephritis (IgAVN) and IgA nephropathy (IgAN)

	IgAVN (*n* = 455)	IgAN (*n* = 160)	*P*
Treatments (n, %)			
Steroid + RASB	236 (51.9)	86 (53.6)	0.682
Steroid + Immunosuppressive agents	57 (12.5)	24 (15.0)	0.426
Methylprednisolone pulse	117 (25.7)	38 (23.8)	0.623
Follow-up time [months, M(1/4,3/4)]	15.5 (6.7,26.5)	15.5 (5.5,31.5)	0.382
Kidney regression (n, %)			< 0.0001
Grade A	282 (62.0)	67 (41.9)	
Grade B	163 (35.8)	73 (45.6)	
Grade C	10 (2.2)	20 (12.5)	
Grade D	0 (0)	0 (0)	

*RASB* renin-angiotensin system blockade

**Table 5:** Risk factors for kidney remission rates in IgA vasculitis nephritis (IgAVN) and IgA nephropathy (IgAN)

Parameter	Univariate				Multivariate			
	IgAVN		IgAN		IgAVN		IgAN	
	HR (95%CI)	*P*	HR (95%CI)	*P*	HR (95%CI)	*P*	HR (95%CI)	*P*
Glomerular lobulation	—	—	1.709 (0.418–6.986)	0.456	—	—	—	—
Mesangial proliferation	20.204 (0–5783939.217)	0.639	1.023 (0.251–4.173)	0.975	—	—	—	—
Cellular crescent	0.706 (0.467–1.066)	0.098	0.552 (0.292–1.044)	0.067	—	—	—	—
Fibrous crescent	1.045 (0.775–1.408)	0.773	1.857 (1.229–2.807)	0.003	—	—	1.138 (0.741–1.747)	0.555
Adhesions	0.817 (0.571–1.167)	0.266	1.518 (0.983–2.343)	0.059	—	—	—	—
Fibrinous thrombosis	—	—	—	—	—	—	—	—
Global sclerosis	1.168 (0.822–1.658)	0.386	0.769 (0.472–1.253)	0.292	—	—	—	—
Segmental sclerosis	0.610 (0.226–1.646)	0.329	1.412 (0.706–2.822)	0.329	—	—	—	—
Thickening of the basement membrane	0.322 (0.102–1.016)	0.053	1.049 (0.498–2.210)	0.901	—	—	—	—
Tubular complete atrophy	1.316 (0.965–1.795)	0.083	5.040 (1.592–15.958)	0.006	—	—	1.561 (0.415–5.863)	0.510
Tubular dilatation	0.974 (0.666–1.425)	0.892	1.068 (0.664–1.718)	0.787	—	—	—	—
Interstitial Fibrosis	1.647 (1.214–2.233)	0.001	4.663 (2.481–8.766)	< 0.0001	1.449 (1.011–2.076)	0.043	2.384 (1.131–5.024)	0.022
Inflammation OR periglomerular inflammation	1.614 (1.088–2.395)	0.017	25.767 (2.074–320.064)	0.011	1.196 (0.758–1.889)	0.441	255896.800 (-)	0.950
Arteriosclerosis OR arterial inflammation	0.751 (0.277–2.033)	0.573	0.999 (0.643–1.551)	0.997	—	—	—	—
Diffuse mesangial proliferation	1.450 (1.076–1.953)	0.015	1.960 (0.714–5.378)	0.191	—	—	—	—
Activity index	0.896 (0.771–1.040)	0.148	0.931 (0.740–1.170)	0.540	—	—	—	—
Chronicity index	1.034 (0.976–1.095)	0.261	1.064 (0.989–1.145)	0.098	—	—	—	—
Total biopsy score	1.017 (0.973–1.064)	0.455	1.046 (0.978–1.119)	0.188	—	—	—	—
IgA +	1.013 (0.516–1.985)	0.971	0.577 (0.287–1.157)	0.121	1.052 (0.536–2.063)	0.883	—	—
IgA + +	2.214 (1.193–4.106)	0.012	1.013 (0.504–2.037)	0.971	2.250 (1.212–4.175)	0.010	—	—
IgA + + +	11.295 (1.442–88.443)	0.021	1.831 (0.232–14.419)	0.566	12.043 (1.498–96.854)	0.019	—	—
C3 +	0.832 (0.556–1.244)	0.370	0.749 (0.455–1.232)	0.255	—	—	—	—
C3 + +	0.988 (0.647–1.477)	0.949	1.278 (0.727–2.247)	0.394	—	—	—	—
C3 + + +	1.452 (0.853–2.471)	0.170	—	—	—	—	—	—

**Fig. 3:**
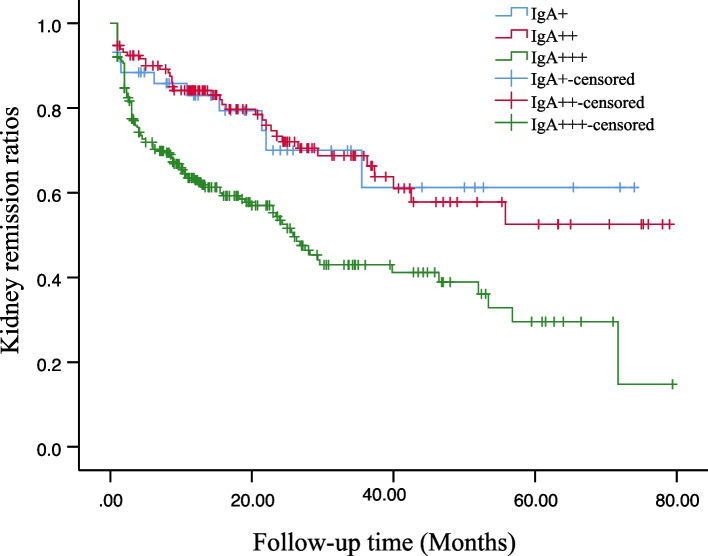
Kaplan–Meier analysis of the effect of IgA deposition intensity on the kidney remission rates of IgA vasculitis nephritis (IgAVN) (Log-rank test, *P* < 0.0001)

The Univariate Cox analysis model revealed associations between interstitial fibrosis, inflammation OR periglomerular inflammation, diffuse mesangial proliferation, intensity of immune complex IgA deposition, and adverse outcomes of IgAVN. Additionally, adverse outcomes of IgAN correlated with the presence of fibrous crescents, complete tubular atrophy, interstitial fibrosis, and inflammation OR periglomerular inflammation. In the multivariate Cox analysis, interstitial fibrosis and IgA +  + and IgA +  +  + appeared to be linked to poor kidney outcomes for IgAVN. For IgAN, only interstitial fibrosis was associated with poor kidney prognosis. Regarding IgAVN, when the intensity of IgA deposition varied, we compared kidney remission rates (Fig. 3). Expectedly, children with IgAVN exhibited significantly lower kidney remission rates when IgA deposition intensity reached +  +  + , indicating a pronounced impact of IgA +  +  + on prognosis.

## Discussion

Our study findings highlighted that both IgAVN and IgAN have a higher prevalence in males, with a relatively larger proportion in IgAN. The SQC chronicity index and total biopsy score were higher for IgAN than for IgAVN, which also exhibited more severe tubular interstitial injury. The deposition intensity of C3, and the rate of IgA pure deposition in the mesangium, were higher in IgAVN than in IgAN, with the intensity of IgA deposition also greater in IgAVN. Notably, IgAVN had a more favorable prognosis and a higher kidney remission rate. According to Cox regression, interstitial fibrosis in the SQC pathology system correlated with the prognosis of both IgAVN and IgAN. Further, IgA +  + and IgA +  +  + might impact the prognosis of IgAVN. Our study also demonstrated a significantly higher incidence of IgAVN during autumn compared to IgAN [21].

Additionally, we noted some significant distinctions; IgAN patients were more prone to gross hematuria than those with IgAVN. Fengmei Wang et al. [22] reported that patients with IgAVN and IgAN have a higher incidence of experiencing left kidney vein compression (nutcracker phenomenon) than those with other kidney diseases. Children with IgAN also reported higher rates of hematuria than those with IgAVN. This correlation warrants further investigation.

Koskela et al. [23] performed serial kidney biopsies on the cohort and assessed the prognostic value of the SQC system for IgAVN in children. They found higher activity scores at diagnostic biopsy, increasing activity and chronicity scores at follow-up biopsy, and that the activity scores declined while chronicity scores increased from the diagnostic biopsy to the follow-up biopsy in most patients, irrespective of proteinuria. The Cox regression results indicated that activity scores at the diagnostic biopsy independently predicted chronicity scores at the follow-up biopsy.

In another study, the authors applied the SQC system to score kidney biopsies in 80 patients with IgAVN and compared the prognostic implications of the International Study of Kidney Disease in Children (ISKDC) and SQC classifications. They found that patients with active kidney disease had higher SQC activity and chronicity scores. The area under the curve was not significantly different between the two at Receiver Operating Characteristic (ROC) curve analysis, demonstrating the effective applicability of the SQC system to patients with IgAVN [24]. A recent multicenter study also suggested that the SQC and Oxford classifications predicted poor kidney prognosis in IgAVN more accurately than the ISKDC classification [25].

In our study, we utilized the SQC system for the first time to assess the severity of kidney lesions in children with IgAVN and IgAN. Compared to the Oxford classification, the SQC system pays more attention to interstitial glomerular lesions. We discovered that pathological scores for complete tubular atrophy, interstitial fibrosis, interstitial inflammation or periglomerular inflammation, and capillary arteriosclerosis or arterial inflammation were higher in IgAN than in IgAVN. Moreover, the chronicity index and total biopsy scores were higher in IgAN, suggesting that kidney pathological changes in IgAN are more severe and demonstrate a chronic pathological course [26]. Our investigation revealed significantly more severe tubulointerstitial lesions in IgAN than in IgAVN, corroborating our findings.

In a retrospective study conducted in Japan, it was discerned that IgAN demonstrated a higher incidence of mesangial proliferation compared to IgAVN [27]. This observation found corroboration in a similar study executed in China [2]. Consistent with these previous findings, our research also showed a more pronounced degree of diffuse mesangial proliferation in IgAN than in IgAVN. The prognostic predictive value of M lesions under the Oxford classification was impacted by the use of immunosuppression, as discovered by Yu et al. [15]. In a similar vein, Shima et al. [28] found that lesion M lost its predictive power in patients undergoing immunosuppressive therapy. In the context of our study, we identified no significant disparity in the utilization rate of immunosuppressants between IgAVN and IgAN. The lack of correlation between mesangial proliferation and kidney prognosis in COX regression univariate and multivariate analyses might be attributed to the analogous mesangial proliferation scores in both groups.

This research found no divergence in the distribution of complex immune types deposited in IgAVN and IgAN. Furthermore, we observed no noticeable differences in the rates of IgG and IgM deposition in the kidneys of IgAVN and IgAN patients, which stood at 40.2% and 38.6%, 73.7% and 74.2% (data not shown), respectively. Mao et al.'s study investigating the clinicopathological relationship between IgAVN and IgAN in children unveiled a striking discrepancy in the blood biochemical parameters of IgAVN and IgAN when the types of immune complex deposition varied [29]. This may be connected to the pathogenesis variability of both conditions. An analysis of the relationship between immune complexes and clinical manifestations remains to be conducted in this paper, and future research is required for more comprehensive understanding.

Our findings indicate significant IgA deposition in the glomerular mesangial zone in both IgAVN and IgAN. Results derived from immunofluorescence deposition intensity revealed that IgA +  +  + predominated in IgAVN, whereas IgA +  + was more prevalent in IgAN. This suggests a higher intensity of IgA deposition in IgAVN, which could be relevant given the primarily acute alterations in the kidney pathophysiology of IgAVN. When a membrane attack complex (MAC) was deposited in the glomerular mesangial zone of IgAVN and IgAN patients' kidneys, alongside IgA and C3 deposits, patients exhibited more severe kidney impairment when MAC was highly expressed, as evidenced by a Canadian study [30]. Consequently, MAC may serve as an independent indicator of kidney injury severity in IgAVN and IgAN patients. However, the relationship between the degree of IgA and C3 deposition and MAC remains to be established. Future investigations are necessary to elucidate their connection to the severity of kidney injury and prognosis. IgAVN and IgAN both demonstrated a predilection for IgA deposition in the glomerular mesangium. In contrast, IgAN exhibited a higher incidence of IgA deposition in both the glomerular mesangium and the segment loop. The increased frequency of IgA deposition in the segment loop might be linked to the more conspicuous capillary alterations associated with IgAN. Furthermore, our study found no discernible disparities in the rates of C1q, C4, and FRA deposition between IgAVN and IgAN.

Our Kaplan–Meier survival analysis unveiled a notably superior prognosis for IgAVN, marked by significantly higher kidney remission rates at 1, 3, and 5 years in comparison to IgAN. As per a specific study, only 30–50% of patients diagnosed with IgAN reach the phase of clinical remission, contrasting sharply with nearly 98% of patients afflicted with IgAVN [31]. These findings align with our research. Through our Cox regression analysis, we ascertained that interstitial fibrosis of the semiquantitative score (SQC) and the strength of IgA deposition may be intertwined with the prognosis of IgAVN, while interstitial fibrosis of SQC was associated with the prognosis of IgAN. Given the comprehensive comparison, interstitial fibrosis emerged as a key determinant influencing kidney outcomes [32–35].

Furthermore, our research identified that cellular crescents were more prevalent in glomerular lesions of IgAVN, implying an acute trajectory of kidney lesions in IgAVN. Huang et al.'s study posited that the severity of kidney pathological presentations in IgAVN escalates with the proportion of crescents [36]. In contrast, our study did not establish a correlation between crescents and the prognosis of IgAVN. The connection between the percentage of crescents and kidney prognosis remains to be elucidated. Wu et al.'s study noted that 6.46% of children with IgAN progressed to kidney failure with cumulative kidney survival rates of 95.3%, 90.3%, and 84% at 5, 10, and 15 years, respectively [37]. Another study highlighted that 20% of children diagnosed with IgAN reached end-stage kidney disease (ESRD) 20 years post-diagnosis, underlining a gradual decline in prognosis in children with IgAN [38]. Given our study's relatively brief follow-up period, none of the children reached the stage of kidney insufficiency.

The strengths of our study include the large sample size, persuasive clinical comparisons, and the novel application of SQC for scoring and accurately assessing kidney pathology in IgAVN and IgAN. Despite these strengths, our study also has limitations: it is a single-center retrospective study, potentially carrying geographical differences and selection bias. Moreover, the data on clinical, laboratory, and treatment parameters are relatively undeveloped, the impact of treatment on pathological tissues remains unclear, the follow-up period is relatively brief, and our prognostic model only incorporates SQC pathological indicators. Future multicenter prospective studies are required to address these limitations.

## Conclusions

Despite certain clinical and pathological similarities, IgAN exhibits more severe kidney impairment, conspicuous interstitial damage, and a poorer prognosis than IgAVN. Significant differences are observed in the intensity and location of immune complex deposition in the kidney. These differences do not support the notion of these two diseases being identical; rather, they suggest two distinct pathologies. The SQC pathological indicator associated with kidney prognosis in children with IgAVN and IgAN could be interstitial fibrosis. The intensity of IgA deposition may be linked to the prognosis of IgAVN.

## Data Availability

The datasets used and/or analyzed during the current study are available from the corresponding author on reasonable request.

## Abbreviations

IgAVN: IgA vasculitis nephritis
IgAN: IgA nephropathy
CI: Confidence intervals
HR: Hazard ratio
IQR: Interquartile range
SQC: Semiquantitative classification
PAS: Periodic Acid-Schiff
PAM: Periodic acid-silver methenamine
RASB: Renin-angiotensin system blockade
MAC: Membrane attack complex
ESRD: End-stage kidney disease
ISKDC: International Study of Kidney Disease in Children
